# On the Use of Haloalkane/Acrylate-Based Holographic Gratings as Compression and Rotation Sensors

**DOI:** 10.3390/s23010183

**Published:** 2022-12-24

**Authors:** Riccardo Castagna, Cristiano Riminesi, Andrea Di Donato, Oriano Francescangeli, Daniele Eugenio Lucchetta

**Affiliations:** 1 URT-CNR@UNICAM, Photonic Materials Laboratory, Università di Camerino (UNICAM), Via Sant’Agostino, 1, 62032 Camerino, Italy; 2 CNR, Institute of Heritage Science, Via Madonna del Piano, 10, 50019 Sesto Fiorentino, Italy; 3 Dipartimento di Ingegneria dell’Informazione, Università Politecnica delle Marche, Via Brecce Bianche, 60131 Ancona, Italy; 4 Dipartimento di Scienze e Ingegneria della Materia dell’Ambiente ed Urbanistica (SIMAU), Università Politecnica delle Marche, Via Brecce Bianche, 60131 Ancona, Italy; 5 Optoacoustic Lab, Dipartimento di Scienze e Ingegneria della Materia dell’Ambiente ed Urbanistica (SIMAU), Università Politecnica delle Marche, Via Brecce Bianche, 60131 Ancona, Italy

**Keywords:** holographic gratings, polymers, acrylate, halo-alkanes, rhodamine 6G

## Abstract

In this work, we test the effectiveness of using highly transparent holographic phase reflection and transmission volume gratings based on multifunctional acrylates as linear compression and rotation sensors. The gratings are recorded in a holographic mixture based on multi-reticulated acrylate and haloalkanes. To activate the photo-polymerization process, we used a mixture of 6-oxocamphore and rhodamine 6G. The mixture is a simplified version of the mixture used in previous works and shows some interesting features mainly in connection with the different roles played by the rhodamine 6G dye at different writing wavelengths λ = 532 nm and λ = 460 nm. Regarding reflection gratings, the maximum achieved diffraction efficiency is ≈50% and their use as linear compression sensors produces a shift in the reflection peak of 2 nm. Following the removal of compression, the grating slowly returns to the initial state. Regarding transmission gratings, the maximum achieved diffraction efficiency is ≈45% and they demonstrate very high sensitivity to even small rotations in a free-standing configuration.

## 1. Introduction

In recent years, the well-established use of holographic gratings as sensors has led to many forms being developed in this research field. Surface relief gratings, inverse opals, metal nanoparticle and nanoparticle-free holographic sensors are used in medical and biological applications [1] or as humidity and temperature sensors [2], whereas flexible substrates are used for the detection of bending deformations [3] and pressure sensors [4]. Regarding pressure sensors, reflection phase holograms recorded in polymer mixtures are used to detect pressure changes by monitoring the reflection peak position as a function of the applied pressure. It is known that, based on the physical and chemical properties of the starting mixture, the less thick materials are suitable for irreversible pressure sensors whereas thicker materials are suitable to design reversible ones [4]. Composite materials are crucial in the fabrication process of displacement and/or pressure sensors. Holographic gratings made by composite polymers represent the most intriguing technological achievement in the field of optical holography over the last few decades [5,6,7,8,9,10,11,12]. An important role in the fabrication of holographic phase gratings is played by multi-acrylate materials that, once polymerized, give rise to a highly interconnected polymer network. The main property of a holographic material resides in the possibility of storing physical information contained in the recording beams. The recorded information can be easily recalled and used in different applications [13,14,15,16,17]. In this work, we investigated the use of recently developed acrylate-based materials for the fabrication of compression and rotation sensors with the aim of incorporating them into other devices such as optically pumped lasers and photomobile polymers [18,19,20]. In the first part of the work, we focus on the possibility of altering the grating pitch by applying external stretching to the grating structure using a linear actuator. In the second part, we focus our attention on the use of free-standing transmission gratings to detect small rotations. To prepare the free-standing samples, we employed a different and simplified experimental approach with respect to the previous published works [3]. The final result shows promise for constructing active sensors based on periodic structures [21,22] or for the application of free-standing holograms to fabricate flexible, stretchable, tunable and switchable devices.

## 2. Materials and Methods

### 2.1. Materials

Dipentaerythritol-hydroxy-penta/hexa-acrylate (DPHA), halo-alkanes (hexyl-bromide (BH) and butyl-bromide (BB)), 6-Oxocamphor (6OC), rhodamine 6G (RH6G) were purchased from Merck, Darmstadt, Germany.

### 2.2. Mixture Preparation

DPHA (≈68%), BH (10%), BB (20%, *w*/*w*), 1.9% 6OC, 0.05% RH6G are blended together until a homogeneous pale orange colour is observed. The mixture is stirred at room temperature, under dark and aerobic conditions for 6 days and then stored for 90 days, in the same environment before being used.

### 2.3. Sample Preparation

The prepared mixture is inserted by capillarity into a sandwich cell made by two microscope glasses separated by two ≈ 50 μm thick mylar strips.

### 2.4. Holographic Recording Set-Up

**Figure 1:**
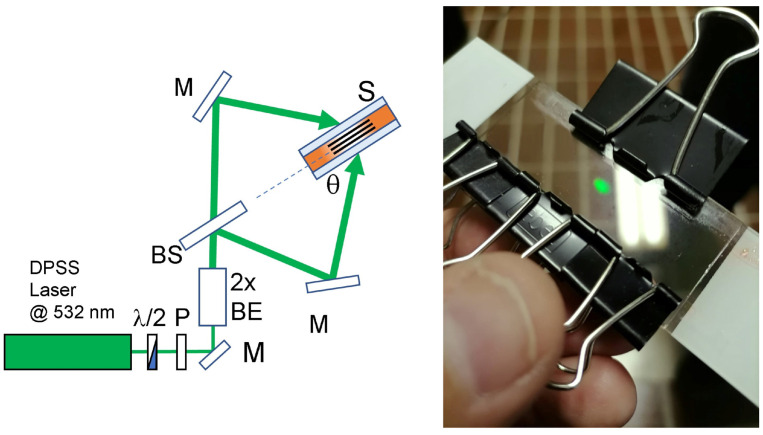
Schematic representation of the writing setup for the high-resolution reflection gratings. λ/2 = half wavelength plate; P = polarizer; M = mirror; 2× BE = 2× beam expander; BS = beam splitter; S = sample; The recorded reflection grating is shown in black. On the right a typical reflection hologram is shown.

To record the reflection phase gratings, we use the experimental setup depicted in Figure 1. The sample is placed in the interfering region generated by the superposition of two s-polarized laser beams emitting a wavelength λ = 532 nm and a power P = 150 mW per beam. The interfering area has a diameter of ≈5 mm while the recording angle θ is ≈54°. The light emitted by the CW green DPSS laser passes through a half-wave (λ/2) waveplate and a linear polarizer (P). This attenuation system allows for the control of the light intensity. After that, the laser beam is expanded by a 2× factor (2× BE) and passes through a beam splitter (BS) which splits the beam on two different mirrors (M). The mirrors redirect the light onto the sample placed in the interfering region. In order to write the transmission grating, we use a single longitudinal mode DPSS laser operating at a wavelength λ = 460 nm having a power P = 150 mW per beam. The recording angle θ is ≈30° measured with respect to the normal to the glass slides. Please note that in order to record a transmission grating, the sample is rotated by 90° with respect to the sample orientation shown in Figure 1. In both situations, the two polymerizing beams impinge symmetrically on the sample (S). Angles are measured in air.

### 2.5. Free-Standing Sample Preparation

After the recording process, the cell is post-polymerized under a UV-A lamp at λ = 365 nm and at a power of P = 0.5 W placed directly in contact with the sample for one minute. After that, the cell is opened and the polymer film containing the grating is peeled-off with the help of a strip of transparent scotch tape. Finally, another strip of the same tape is applied on the other side of the film to seal the entire system. At this stage, the free-standing film is ready for use.

### 2.6. Reading Set-Up

The reading setup consists of a white Xe-lamp that illuminates the sample through an optical fiber (OFS) while an external compression is applied to the cell using a clamp (C). Changes in the reflected signal are monitored by using an optical fiber (OFD) connected to a real-time spectrometer (SP). Detection of the diffracted signal on free-standing samples is achieved by applying rotation to the film containing a transmission grating while a white Xe-lamp-generated light illuminates the sample. The signal transmitted by the grating is acquired through an optical fiber connected to a real-time spectrometer. For each rotation angle $\theta_{r}$, part of the impinging light is Bragg diffracted. In other words, the signal acquired by the SP will show a hole corresponding to the narrow band of frequencies diffracted for that specific rotation angle. Depending on the direction of rotation, we observe either a red- or blue-shift of the diffracted wavelengths.

## 3. Results and Discussion

**Figure 2:**
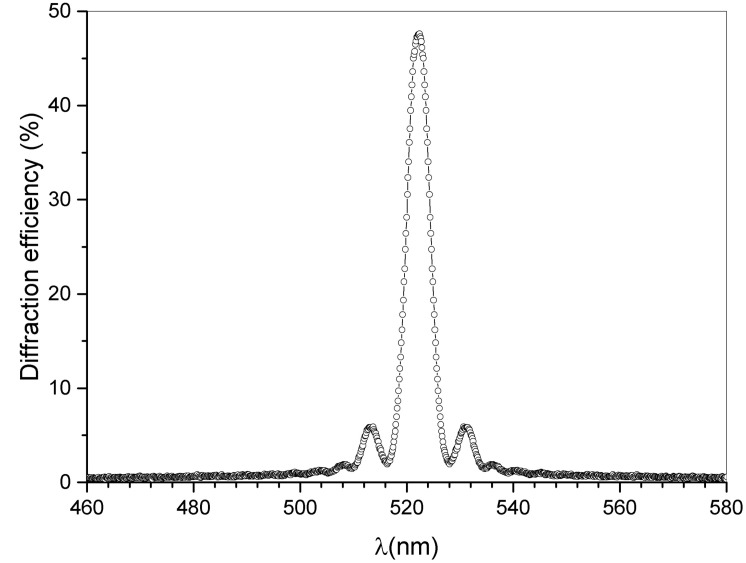
Typical normalized sample transmission measured using white light, impinging perpendicularly to the glass slides, and connected to a spectrometer.

The calculated average refractive index of the grating is n = 1.49. The grating pitch at the end of the writing and post-polymerization process for the reflection holograms is Λ ≈ 175 nm, while the grating pitch for the transmission grating is Λ≈ 450 nm. Values are measured at the end of the post-polymerization and shrinkage processes [23]). A typical normalized transmission spectrum regarding a reflection grating recorded with the setup reported in Figure 1 is shown in Figure 2. In this case, a Xenon white light impinges perpendicularly on the glass slides containing the reflection grating and the result is a narrow notch with a depth that directly represents the diffraction efficiency of the grating itself. During the recording process at $\lambda_{w}$ = 532 nm, absorption of the photoinitiator OC is maximum in the blue-region of the electromagnetic spectrum, while the rhodamine 6G absorbs in a wavelength region between 500 and 550 nm, with the maximum at λ = 530 nm [24]. It is reasonable to hypothesize a synergistic effect in the photo-initiation that involves an electron-transfer process and a proton-transfer with consequent production of free radicals able to start the photopolymerization.

**Figure 3:**
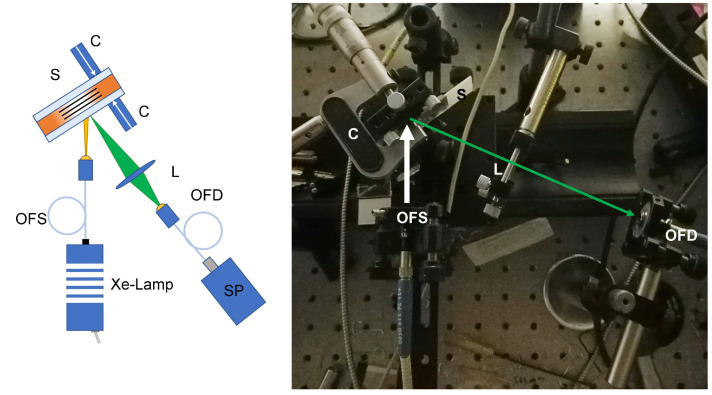
Reading setup. OFS: optical fiber (light) source; S: sample; C: clamping system; L: lens; OFD: optical fiber (light) detector; SP: Spectrometer. Shown on the right side is the real experimental setup.

Values around ≈50% were obtained showing a remarkable FWHM of 5 nm, using incoherent white light. This measured FWHM value shrinks by 0.7 nm when full compression is applied to the sample. The corresponding high value of angular selectivity represents an optimal feature for the detection of small-wavelength shifts due to the application of compression on both sides of the cell and near the grating area. Figure 3 shows the setup used to detect the reflected signal as a function of the applied compression. A micrometric clamp acts near the spot area and an optical fiber detector (OFD) placed on the reflected signal is used to monitor any possible change in the diffracted signal due to the applied linear compression.

**Figure 4:**
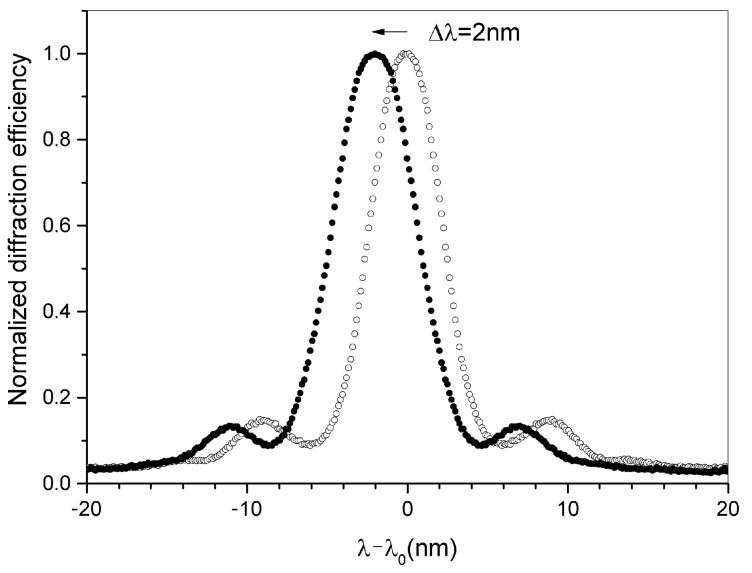
Normalized diffraction efficiency as a function of the wavelength difference λ-$\lambda_{0}$ before (empty circles) and after full compression (black-filled circles), $\lambda_{0}$ is the wavelength reflected by the grating without compression.

As reported in Figure 4 at the maximum of the applied displacement, the measured wavelength shift is approximately 2 nm.

**Figure 5:**
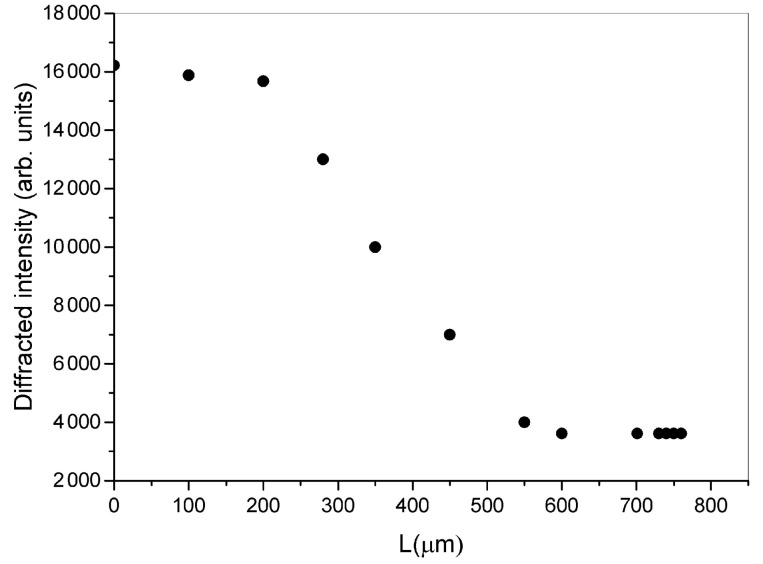
Diffracted intensity as a function of the linear applied compression on the two sides of the cell.

At the same time, the grating diffraction efficiency decreases to a minimum value as shown in Figure 5. This means that even such a small induced displacement can be detected by carefully checking the diffraction efficiency values.

**Figure 6:**
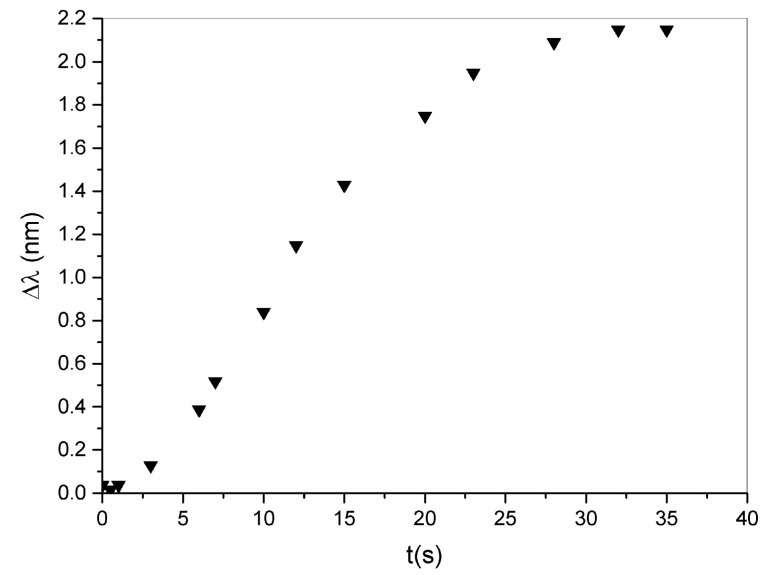
Wavelength shift as a function of time. By removing the compression, the system returns to the initial state.

By completely removing the applied force, the system gradually returns to the initial condition as reported in Figure 6.

**Figure 7:**
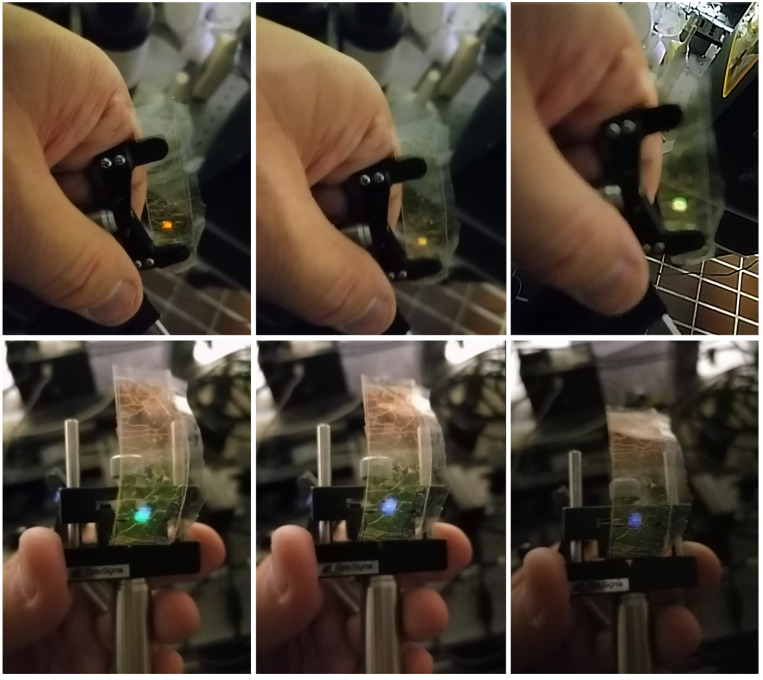
A typical free-standing sample, the colours of which change by altering the angle of vision with respect to the ambient light. Colours shown range from deep red to deep blue.

Unfortunately, the time needed to restore the initial condition limits the use of our halo-alkane/acrylate-based mixture as compression sensors. As a consequence, we decided to test the device in reflection mode and in a free-standing configuration. The free-standing films are prepared by following the procedures given in Section 2.3. We also decided to use a different writing wavelength in order to avoid the rhodamine 6G absorption band. The recorded grating on the free-standing support shows different colours when the room light illumination angle is altered, as shown in Figure 7.

**Figure 8:**
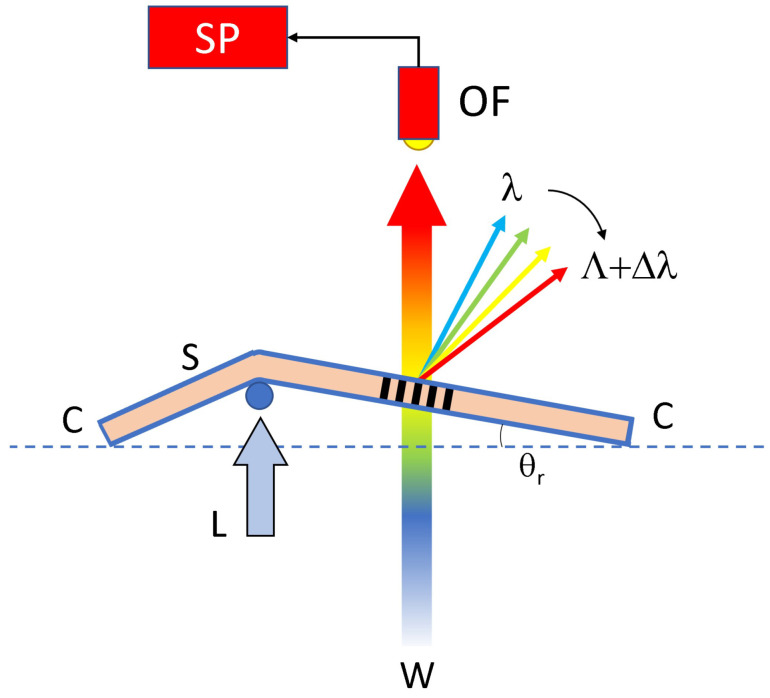
Setup used to read the wavelength changes as a function of the rotation angle $\theta_{r}$. By increasing the applied displacement L, the angle increases and the transmission grating selects different wavelengths ranging from light blue to deep red. C = clamping; S = Sample; OF = Optical Fiber; W = White light source; SP = Spectrometer.

The free-standing film containing the transmission grating is clamped on the two edges and subjected to an external linear displacement (L) by using a small vertical rod, the section of which is represented in Figure 8 as a small blue circle. As a consequence of the applied displacement, the sample rotates by a few degrees. The rotation angle $\theta_{r}$ is the quantity we measure in this experiment. The grating is illuminated by a white light and the sample transmission is monitored through an optical fiber (OF) connected to a real-time spectrometer. For each value of the rotation angle $\theta_{r}$, a narrow range of wavelengths impinging on the grating is diffracted at their corresponding Bragg angles. The diffracted wavelengths range from light blue to deep red. A red or blue shift can be easily obtained by applying displacement on the left side of the grating or on the right side, respectively.

**Figure 9:**
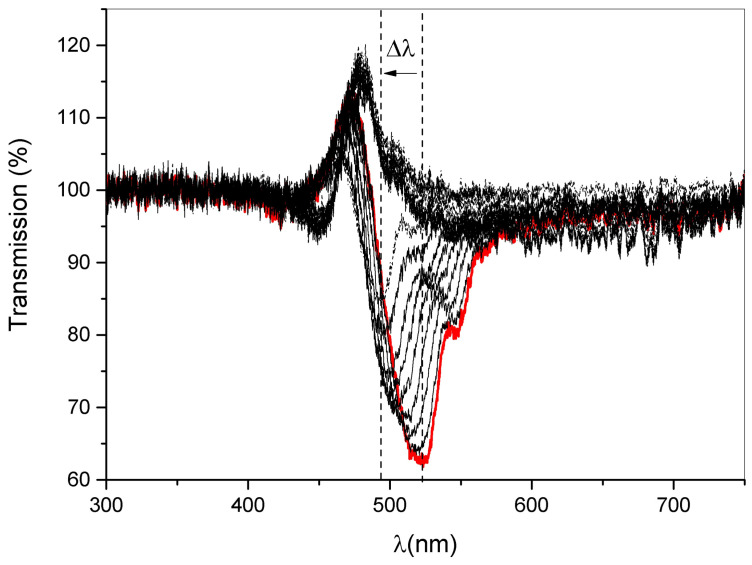
Behaviour of the transmission peak as a function of the wavelength. The reflection notch has a blue shift Δλ = 30 nm when a rotation of 0.2 rad is applied to the free-standing film. The red curve represents the start of our measurement.

The changes in diffracted wavelengths are monitored in real time by acquiring the transmission spectra of the sample at different rotation angles. A typical result is reported in Figure 9. As expected, the notch corresponding to the reflected light has a blue shift of 30 nm when a rotation of 0.2 rad is applied to the free-standing film.

**Figure 10:**
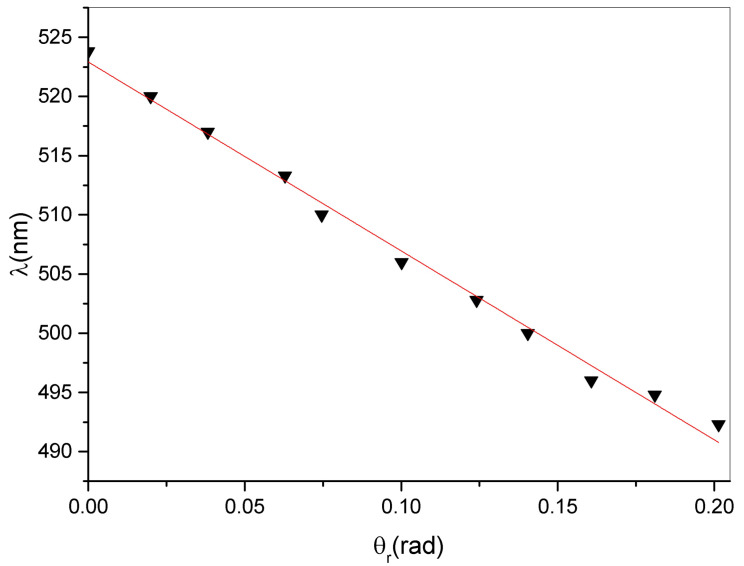
Position of the minimum of the reflected peak as a function of the rotation angle $\theta_{r}$.

Finally Figure 10 shows the changes in minimum peak position as a function of the calculated rotation angle $\theta_{r}$. A simple linear regression gives a slope of 159 ± 4 nm/rad which represents the sensitivity of our measurement system [3]. In principle, these data can be coupled with a reduction in diffraction efficiency in order to reduce the error in the measured values of rotation angle.

## 4. Conclusions

In conclusion, we investigated the use of a haloalkane–acrylate mixture as a compression and rotation sensor. The mixture is a simplified version of a mixture used in previous works and in different applications. Reflection and transmission gratings were recorded and employed to detect small compressions and rotations. Transmission volume holographic gratings are used in a free-standing configuration producing sensitivity values up to 159 nm/rad. We believe that these specific gratings can find application in fields requiring detection of small compressions and rotations.

## Data Availability

Data are available from the authors upon reasonable request.
